# Prevalence of Job Strain Among Assembly Line Workers

**DOI:** 10.1192/j.eurpsy.2026.11846

**Published:** 2026-07-31

**Authors:** F. Dhouib, A. Kchaou, N. Kotti, N. Remadi, M. Hajjaji, K. Jmal Hammami

**Affiliations:** Occupational medecine Department, Faculty of medecine of Sfax, Sfax, Tunisia

## Abstract

**Introduction:**

Workers in assembly line work are exposed to high levels of physical strain, mental fatigue, and limited autonomy over their tasks. These demanding conditions can lead to psychological distress, reduced job satisfaction, and an increased risk of burnout or other stress-related disorders.

**Objectives:**

The objective of this study was to assess the prevalence of job strain among assembly line workers.

**Methods:**

We conducted a descriptive cross-sectional study among assembly line workers in a cosmetics packaging unit. Data collection was based on the Karasek Job Content Questionnaire, supplemented with individual employee information.

**Results:**

Our study included 26 workers, consisting of 24 women and 2 men, with a mean age of 35.1 years. The majority were married (61.5%). Most participants had a secondary education level (96%), while only 4% had higher education. About half of the population (46%) reported at least one medical history, including hypertension in 11.5% of cases and psychiatric disorders in 3.8%. Nineteen percent suffered from musculoskeletal disorders.

With regard to occupational characteristics, the average job tenure was 10.11 years. Nearly half (46.1%) had previously held another position. Seventy-three percent of workers were assigned to afternoon shifts, 3.8% to night shifts, and the remaining 23.2% to morning shifts.

A high psychological job demand was reported in 80.7% of cases. Job strain was identified in 65.3% of participants, while isostrain was observed in 46.1%

**Conclusions:**

Monotony is a defining feature of assembly line work and represents a source of occupational stress, regardless of other socio-professional risk factors. Occupational physicians should pay particular attention to assembly line workers, who constitute a vulnerable population to job-related stress. Such stress can have long-term health consequences, including an increased risk of cardiovascular disease, recurrent musculoskeletal disorders, and anxiety-depressive symptoms.

**Disclosure of Interest:**

None Declared

